# The legacy of hope summit: a consensus-based initiative and report on eating disorders in the U.S. and recommendations for the path forward

**DOI:** 10.1186/s40337-021-00501-w

**Published:** 2021-11-04

**Authors:** Donald Blackwell, Carolyn Becker, Ovidio Bermudez, Michael E. Berrett, Gayle E. Brooks, Douglas W. Bunnell, Dena Cabrera, Carolyn Costin, Nancy Hemendinger, Craig Johnson, Kelly L. Klump, Cheri A. Levinson, Michael Lutter, Margo Maine, Carrie J. McAdams, Beth Hartman McGilley, Stuart B. Murray, Elissa Myers, J. D. Ouellette, Christine M. Peat, Kristina Saffran, Stephanie Setliff

**Affiliations:** 1 Bowman and Brooke LLP, Lake Mary, FL USA; 2 Trinity University, One Trinity Place, San Antonio, TX 78212 USA; 3 Child and Adolescent Services, Eating Recovery Center and Affiliates, 7351 Lowry Blvd., Suite 200, Denver, CO 80230 USA; 4 Center for Change, Berrett and Company, LLC, 1790 N State Street, Orem, UT 84057 USA; 5 The Renfrew Center of Florida, 7700 Renfrew Lane, Coconut Creek, FL 33073 USA; 6 Westport, USA; 7 Clinical Services, Rosewood Centers for Eating Disorders, 950 West Elliot Road, Suite 201, Tempe, AZ 85284 USA; 8 Carolyn Costin Institute, 29066 Cliffside Dr., Malibu, CA 90265 USA; 9 Office of Health Education, Suffolk County Department of Health Services, Office of Health Education/Division of Preventive Medicine, North County Complex, Veteran’s Memorial Highway, Bldg. 016, Box 6100, Hauppauge, NY 11788 USA; 10 Family Institute, Eating Recovery Center, 7351 Lowry Blvd., Suite 200, Denver, CO USA; 11 MSU Foundation, Michigan State University Twin Registry, Department of Psychology, Michigan State University, 316 Physics Road – Rm. 107B, East Lansing, MI 48824 USA; 12 Eating Anxiety Treatment Laboratory, Behavioral Wellness Clinic/Louisville Center for Eating Disorders, Department of Psychological and Brain Sciences, University of Louisville, Louisville, KY USA; 13 Eating Recovery Center of San Antonio, Precision Psychiatry, 8105 Rasor Blvd #235, Plano, TX 75024 USA; 14 Maine and Weinstein Specialty Group, LLC, 433 South Main Street, Suite 327, West Hartford, CT 06110 USA; 15 Psychiatry With Tenure, Brain-Body Perceptions Research Program, UT Southwestern Medical Center, 6363 Forest Park Road, BL 6.204, Dallas, TX 75390 USA; 16 UKSM-W (Volunteer), 10500 E Berkeley Square Pkwy St 103, Wichita, KS 67206 USA; 17 Eating Disorders Program, Translational Research in Eating Disorders Laboratory, Department of Psychiatry and Behavioral Sciences, USC Keck School of Medicine, University of Southern California, 2250 Alcazar Street, Suite 2200, Los Angeles, CA 90033 USA; 18 Academy for Eating Disorders, 11130 Sunrise Valley Drive, Suite 350, Reston, VA 20191 USA; 19 Peer Coaching, University of California San Diego Center for Eating Disorders Treatment and Research, 11175 Negley Ave., San Diego, CA 92131-1819 USA; 20 National Center of Excellence for Eating Disorders, Psychiatry, University of North Carolina at Chapel Hill, CB #7160, Chapel Hill, NC 27599-7160 USA; 21 Equip Health, Project Heal, 2674 Costebelle Drive, La Jolla, CA 92037 USA; 22 Eating Recovery Center, 4708 Alliance Blvd, Suite 300, Plano, TX 75093 USA

**Keywords:** Anorexia nervosa, Bulimia nervosa, Binge-eating disorder, Avoidant/restrictive food intake disorder, Advocacy, Standards, Body image

## Abstract

**Background:**

Several unsuccessful attempts have been made to reach a cross-disciplinary consensus on issues fundamental to the field of eating disorders in the United States (U.S.). In January 2020, 25 prominent clinicians, academicians, researchers, persons with lived experience, and thought leaders in the U.S. eating disorders community gathered at the Legacy of Hope Summit to try again. This paper articulates the points on which they reached a consensus. It also: (1) outlines strategies for implementing those recommendations; (2) identifies likely obstacles to their implementation; and (3) charts a course for successfully navigating and overcoming those challenges.

**Methods:**

Iterative and consensual processes were employed throughout the Summit and the development of this manuscript.

**Results:**

The conclusion of the Summit culminated in several consensus points, including: (1) Eating disorder outcomes and prevention efforts can be improved by implementing creative health education initiatives that focus on societal perceptions, early detection, and timely, effective intervention; (2) Such initiatives should be geared toward parents/guardians, families, other caretakers, and frontline healthcare providers in order to maximize impact; (3) Those afflicted with eating disorders, their loved ones, and the eating disorders community as a whole would benefit from greater accessibility to affordable, quality care, as well as greater transparency and accountability on the part of in-hospital, residential, and outpatient health care providers with respect to their qualifications, methodologies, and standardized outcomes; (4) Those with lived experience with eating disorders, their loved ones, health care providers, and the eating disorders community as a whole, also would benefit from the establishment and maintenance of treatment program accreditation, professional credentialing, and treatment type and levels of care guidelines; and (5) The establishment and implementation of effective, empirically/evidence-based standards of care requires research across a diverse range of populations, adequate private and government funding, and the free exchange of ideas and information among all who share a commitment to understanding, treating, and, ultimately, markedly diminishing the negative impact of eating disorders.

**Conclusions:**

Widespread uptake and implementation of these recommendations has the potential to unify and advance the eating disorders field and ultimately improve the lives of those affected.

**Plain English summary:**

A cross-disciplinary group of eating disorder professionals, thought leaders, and persons with lived experience have come together and reached a consensus on issues that are fundamental to the battle against the life-threatening and life-altering illnesses that are eating spectrum disorders. Those issues include: (1) the need for early detection, intervention, prevention, and evidenced-based standards of care; (2) the critical need to make specialized care more accessible and affordable to all those in need; (3) the importance of developing uniform, evidenced-based standards of care; (4) the need for funding and conducting eating spectrum disorder research; and (5) the indispensability of advocacy, education, and legislation where these illnesses are concerned. During the consensus process, the authors also arrived at strategies for implementing their recommendations, identified likely obstacles to their implementation, and charted a course for successfully navigating and overcoming those challenges. Above all else, the authors demonstrated that consensus in the field of eating spectrum disorders is possible and achievable and, in doing so, lit a torch of hope that is certain to light the path forward for years to come.

## Background

In the summer of 2019, the father of a young woman,^1^ who spent nearly a decade locked in a life-and-death struggle with an eating disorder, saw the need for and the benefits that likely would flow from a consensus-based approach to issues fundamental to the eating disorder battle—a goal that has eluded the U.S. eating disorder community for more than four decades. It was then that the idea of a summit involving a diverse group of some of the most widely-respected eating disorder researchers, academicians, clinicians, thought leaders, advocates, and persons with lived experience in the U.S. was conceived. Invitations were sent and, in January 2020, more than two dozen invitees convened—each at their own expense—for the Legacy of Hope Summit.

The goals of the Summit were ambitious: (1) to articulate and reach a consensus regarding a series of recommendations on issues relating to: (a) prevention, early detection, and early intervention; (b) accessibility, affordability, and accountability; (c) standards of care; (d) research and research funding; and (e) advocacy, education, and legislation; that all believed would have a significant beneficial impact on those afflicted with eating disorders, their loved ones, and the eating disorders community as a whole; (2) to develop short and long term strategies for implementing those recommendations; (3) to identify likely obstacles to their implementation; and (4) to chart a course for successfully navigating and overcoming those challenges.

All acknowledge that there is much work still to be done and that there is room to disagree over a word here or a phrase or sentence there. Moreover, the authors realize that not everyone in the field will agree with everything in this paper. However, given the gravity of the situation and the preciousness of the lives hanging in the balance, the consensus is that: (1) the status quo is unacceptable; (2) the need for a thoughtful and unified plan of action is immediate; and (3) the time for meaningful progress is long overdue. Thus, the authors’ and the supporting endorsers’ hope is that this paper will be a living document that will serve as a catalyst for further consensus-building and an initial blueprint for hope and healing for years to come.

## Methods

Participants in the Summit were chosen by the initiative’s organizer with the aim of achieving a balanced cross-section of the various stakeholders in the eating disorder community (e.g., clinicians providing treatment at all levels of care (i.e., from outpatient to inpatient and residential), researchers, thought leaders, advocates, academicians, and persons with lived experience). It is noteworthy that the invitees and eventual attendees included a number of current or former founders, board members, and/or executive directors of major eating disorders organizations, including AED, iaedp, NCEED, NEDA, and Project Heal. Invitations also were informed by the organizer’s nearly decade long involvement in and familiarity with a wide variety of experts in the eating disorders community, as well as recommendations from several widely-respected leaders in the field. Moreover, to help foster dialogue, the desired total number of participants was set at approximately 25, so that each of the five work groups would be populated with five members.

The Summit participants, in turn, were assigned to five work groups—one for each of the Consensus Points outlined in this paper according to their stated preferences and their areas of expertise. However, in order to build a true consensus and derive the full benefit of the attendees’ considerable and broad-based experience, each participant also was afforded an opportunity to provide input on any or all of the other work groups in advance of the Summit, and many did so.

Each work group was then provided with a packet of materials that included: (a) a proposed Consensus Point; (b) a working draft of the “status quo” relating to their assigned subject area; and (c) an outline of the five areas referenced in the Background section (above). They, in turn, were challenged to engage in open and vigorous debate on each of those documents and subject matters until they arrived at a consensus within the work group.

At the conclusion of the Summit, a timetable was established and agreed upon for moving the initiative forward and each work group was asked to designate a leader, whose roles included finalizing their group’s report and serving as the point person for future communications with the group. In the ensuing three months, each of the work groups completed, vetted, approved, and submitted their individual reports. Those reports were then woven into a single document before being circulated to all Summit attendees for their review, comment, and approval.

Over the next six months, the resulting document went through a series of revisions within the individual work groups, before being circulated a second time to the Summit participants for their final review and approval. Once a unanimous consensus was reached, each of the Summit participants was encouraged to send the draft Report to colleagues and other principal stakeholders in the eating disorders field, as well as those with lived experience, for their review and support. The resulting Report, which forms the basis for this paper, is the culmination of those efforts and represents the collective wisdom and consensus recommendations of all Summit participants and endorsers.

## Results

The results of the Summit are organized by work group as below:

- *Section I* Prevention, Early Detection, and Intervention
- *Section II* Accessibility, Affordability, and Accountability
- *Section III* Standards of Care
- *Section IV* Research and Research Funding
- *Section V* Advocacy, Education, and Legislation

Each section contains an overview of the status quo for each topic area, a Consensus Point, and a list of goals and strategies. The results here are intended to serve as a practical roadmap for improving and advancing the eating disorders field as a whole.

### Section I: prevention, early detection, and intervention

*The Status Quo* Various presentations of eating disorders occur in people from all walks of life across the lifespan, yet they remain under-detected and under-treated [1]. Despite the fact that there is increasing evidence that school-based eating disorder screening at primary, middle, and secondary school levels is as effective as other health-based screenings in reducing the dollars spent and years lost in later treating and battling those disorders (not to mention the impact they have on quality of life), such screenings and referrals for early intervention are not routinely done in U.S. schools at any level. In fact, there has never been an organized national screening program in place in school or primary healthcare settings for pre-adolescents. Further, in 2013, the Centers for Disease Control (CDC) removed several questions from the National Youth Risk Behavior Survey (YRBS) that had provided surveillance for those engaging in disordered eating behaviors.

Preschool through secondary school comprehensive health education (including a focus on health literacy, adopting healthy behaviors, and valuing wellbeing) with a parent component provides the opportunity for increasing knowledge and skills to act in healthy ways and builds personal value for healthy behaviors at home and in schools. Although preschool through secondary school comprehensive health education is mandated in most states, there are no formal assessments, as there are with other subjects like math and language arts. As a result, health education is fit into curriculums as an afterthought and not a priority. In addition, there is not uniform preschool through secondary school health education teacher training to enable teachers to confidentially address sensitive health topics and how to build a healthy norm within the classroom. In addition, prevention efforts in adults are almost non-existent with the exception of programs that focus on college students. The lack of education to healthcare providers exacerbates this problem among children, adolescents, and adults.

The same conundrum exists in the areas of early detection and treatment intervention. More specifically, there is an evolving body of medical and scientific literature indicating that both can have a meaningful impact on eating disorder sufferers’ symptom severity, quality of life, and mortality rate, and yet, disturbingly, few individuals with eating disorders across the diagnostic spectrum receive treatment [2]. Even more troubling, symptoms that could lead to early detection and intervention are often missed in atypical presentations, males, communities of color, and people with body types and weights that are not commonly perceived to be associated with eating disorders. Additionally, there exists an under recognition of the complex psychiatric (e.g., mood disorders, non-suicidal self-injury, and suicide risk) and medical comorbidities (e.g., cardiac, metabolic, endocrine, etc.) associated with eating disorders.

Although universal prevention is ideal, there is some debate as to whether it is achievable. Evidence from targeted prevention efforts, however, is convincing many that universal prevention is possible [3]. To date, however, little has been done to implement and disseminate prevention initiatives and there is a considerable amount of work still to be done in estimating the willingness and the cost associated with their implementation.

*Consensus Point*: Eating disorder outcomes and prevention efforts can be improved by implementing creative health education initiatives that focus on societal perceptions, early detection, and timely, effective intervention. Such initiatives should be geared toward parents/guardians, families, other caretakers, and frontline healthcare providers in order to maximize impact (Table 1).

**Table 1:** Goals and strategies for prevention, early identification, and intervention

Goal	Strategy	Obstacles	Navigation
*Prevention*			
Implement comprehensive health education programs that meet the National Health Education Standards (from the CDC) and include culturally-appropriate information that focuses on social-emotional development, enhancement of protective factors, and establishment of healthy peer norms	Use legislative efforts to enforce mandates and measurements for state education departments	Not every state can or will enforce/adopt the mandates due to: Limited resources Lack of recognition and prioritization of eating disorders Lack of collaborative effort focused on early detection and prevention between the public and mental health disciplines	Nominate/identify a group that lobbies for these initiatives Build grassroots support and understanding for the importance of implementing preschool through 12th grade comprehensive health education with built in assessments Use other health indicators such as dietary patterns, diabetes, and mental illness rates as a way to build support for eating disorder screenings and implementation of preschool-secondary school comprehensive health education Develop a mechanism for cross-discipline dialogue between public and mental health professionals using easy-to-implement, low cost programs (e.g., existing technology programs)^a^
*Early identification and intervention*			
Recognize risk behaviors, at-risk statuses, early development of the illnesses in typical and atypical clinical presentations; and appropriately intervene and/or refer	Broadly disseminate evidence-informed content, strategies, and tools via NCEED, a nationally-recognized, not-for-profit organization with the ability to reach a diverse group of stakeholders	There will likely be difficultly in adequately reaching all stakeholders who might play a role in detection and early intervention. This is particularly true for primary care and frontline providers as they already are heavily burdened with screening a variety of mental and physical health conditions	Key partnerships with organizations will help promote strategy (e.g., ACCME; Boards of primary care specialties; the CDC; teachers’ unions; state education departments; NASMHPD; etc.)^b^
Implement developmentally, age, gender, race, culturally-appropriate screening practices in primary care and ambulatory care settings	Preschool—secondary school and public colleges and universities: Engage with legislative bodies to enact legislation that compels providers (and other stakeholders) at publicly-funded institutions to receive education and training on eating disorder detection and early intervention Adults: Develop standards of practice for screening and early intervention and/or leverage the power of electronic medical records (e.g., Epic) to help providers engage in this process. For example, an electronic medical record/clinic workflow might include a brief screening for eating disorders which then triggers specific steps and/or referrals for patients at high risk^e^	Lack of awareness or buy-in from primary care providers and other frontline clinicians who may see screening for yet another condition as an additional burden; viewing eating disorders as a low priority concern; prioritization of addressing “obesity problem” overeating disorders; and general lack of understanding about the screening process	Identify and use influencers and/or consensus building organizations^c,d^
Include eating disorder-informed content into existing higher education and workplace wellness initiatives (e.g., employee-based programs that promote improving dietary and physical activity patterns, stress reduction, mindfulness practices, etc.)	Promote the cost-saving value of the wellness initiatives	Lack of awareness or buy-in from employers/companies, schools, and organizations	Use influencers to encourage change from within corporate governance Sell as a quality improvement in the workplace (lower cost associated with health insurance; increased importance in value-based care, etc.) Highlight the cost to employers from absenteeism, turnover, etc. of undiagnosed or untreated eating disorders

^a^Important Contacts: Department of Health and Human Services; National Eating Disorders Association; Eating Disorders Coalition; State Boards of Education; Superintendent’s Associations; and Parent Teacher Associations

^b^Important Contacts: National Center of Excellence for Eating Disorders; National Eating Disorders Association; Academy for Eating Disorders; International Association of Eating Disorder Professionals; Eating Disorder Coalition; State Medical Boards

^c^As with many other points in this document, more work will need to be completed to determine how to make this happen

^d^Important Contacts: Thinktank of people who represent various stakeholder groups and have knowledge in these types of processes (e.g., smoking cessation; depression screening, etc.)

^e^To this end, NCEED was recently granted $300,000 in supplemental funding by the Substance Abuse and Mental Health Services Administration (SAMHSA); that provided the initial funding to establish NCEED to develop a primary care-specific protocol for detection and management of eating disorders with an eye toward leveraging the power of electronic medical record systems. This protocol will equip frontline clinicians with the necessary training and tools to engage in early detection and intervention for eating disorders

*ACCME* Accreditation Council for Continuing Medical Education, *CDC* Centers for Disease Control, *NASMHPD* National Association of State Mental Health Program Directors, *NCEED* National Center of Excellence for Eating Disorders

### Section II: accessibility, affordability, and accountability

*The Status Quo* Eating disorders are treatable illnesses, and full recovery is possible given access to quality care for the requisite period of time. However, too few patients have access to timely evaluation and/or the appropriate level and duration of care required to achieve and sustain full recovery [4]. A number of factors contribute to this state of affairs, including: (1) the prohibitive cost of treatment at every level of care; (2) health insurers’ refusal to reimburse or adequately reimburse for the required care; (3) the disparity between what is covered by private and government funded insurance; (4) biases related to a narrow perception of the type of person who is most likely to struggle with an eating disorder; and (5) the relative scarcity of eating disorder providers and support resources, especially in underserved populations and areas [5].

Lack of access to expert evaluation and treatment for eating disorders is especially prevalent in populations that do not conform to existing stereotypes [6]. Thus, it is vital that we develop models of education, early identification, and support that effectively engage and support all at-risk populations.

Lastly, accountability by providers at all levels of care is essential. Relapse rates appear exceedingly high but are difficult to quantify because those in a position to do so (e.g., residential treatment providers) rarely report short and long-term outcomes for the treatments they provide and there is no empirically-derived, consensus-driven definition of recovery with which to evaluate outcomes.

To address the foregoing gaps in access to expert care (e.g., the shortage of providers with specialized training [7] and their geographic dispersion, the enormous financial and public and private insurance barriers, and the variability in the information, treatment recommendations, and care offered by specialized and non-specialized providers, etc.), as well as to demonstrate treatment effectiveness, the eating disorders field must strive to ensure that all impacted populations are: (1) properly screened and identified utilizing consistent and standardized protocols; (2) educated on evaluating treatment options grounded in evidence-based practices; and (3) afforded access to appropriate levels and quality of care. We believe these are the essential components to obtain full recovery—and that they are achievable.

*Consensus Point* Those afflicted with eating disorders, their loved ones, and the eating disorders community as a whole would benefit from greater accessibility to affordable quality care, as well as greater transparency and accountability on the part of in-hospital, residential, and outpatient healthcare providers with respect to their qualifications, methodologies, and standardized outcomes (Table 2).

**Table 2:** Goals and strategies for accessibility, affordability, and accountability

Goal	Strategy	Obstacles	Navigation
Establish true partnerships between clients and their families that address individualized treatment needs while working within the framework of uniform treatment standards Establish empirically derived consensus definitions of recovery that are inclusive for all patients and practical for both research and treatment settings Establish field consistency and transparency on collection and dissemination of data and outcomes, including over time Establish comprehensive, multidisciplinary education materials on eating disorders for relevant training programs (MDs, PhDs, MSWs, RDs, etc.) Emphasize care provided within the community Develop nationally accepted, empirically supported standards designed to accurately quantify patient progress Develop a consensus approach on methods for assessing readiness for change (independent adult populations) Remove gender specific criteria for admission at all levels of care	Research into barriers to treatment access for individuals with eating disorders in the U.S^a^ Training of specialized providers with comprehensive, multidisciplinary education materials on eating disorders for relevant training programs (MDs, PhDs, MSWs, RDs, etc.) broadly disseminated via NCEED Prioritization of advocacy efforts to address the lack of public funding for eating disorders treatment Establishment of field consensus on treatment standards, including core components of treatment at every level of care with consideration of cultural differences (i.e., to the extent practicable, treatment standards and venues should account/allow for the full spectrum of eating disorder patients, including different dietary needs, family structures, gender expressions, religious faiths, body weights etc.) Establishment of program standards that ensure each patient and family has been provided clear expectations about the current research on the treatment of eating disorders. This standard of “true informed consent” would also include the rationale, if applicable, for recommending treatments that do not have strong research support	Lack of training for non-professional caregivers, medical providers, and graduate level clinicians^b^ Failure to provide clients/families with descriptions of the full nature of eating disorders treatment and recovery Cultural incompetence and associated issues related to working with underserved populations Lack of Medicaid/Medicare coverage Failure of third-party payors to reimburse in a timely manner and at an appropriate rate relative to the provider’s and/or facility’s level of expertise/level of care Lack of consensus about even the basics of eating disorders care at higher levels of care (e.g., establishing weight ranges, defining weighing protocols, etc.)	In situations where medical and psychiatric stability are present, use low-intensity interventions related to screening, early identification, use of online resources, and guided self-help Training of non-professionals to provide peer support or coaching Nutritional psychoeducation via apps and other online support mechanisms Use of algorithms to inform treatment and level of care decisions Virtual treatment/teletherapy at all levels of care True informed consent: a statement read by all providers/centers outlining all options Increased funding for research focused on marginalized communities (i.e., underserved populations) with eating disorders so that the field can both understand their needs and develop strategies to address those needs
	Using nationally accepted, empirically-supported standards designed to accurately quantify patient progress, AED, REDC, and other prominent advocacy/professional organizations should mandate and support data collection and publication using common metrics Establishment of empirically-derived consensus definitions of recovery that are inclusive for all patients and practical for both research and treatment settings The development of a consensus approach on methods for assessing readiness for change for adult patients with eating disorders Designing, studying, and implementing innovative treatment programming that emphasizes care in the community. The field should also support the study and use of technology to extend access to treatment opportunities Improving access to care for marginalized communities with eating disorders by removing gender-specific criteria for admissions at all levels of care and by identifying, training, and hiring more people who reflect the full spectrum of eating disorder sufferers		Hire more people who reflect the full spectrum of eating disorders sufferers (i.e., who mirror the racial, ethnic, size, cultural diversity, gender identity, and sexual orientation of those who suffer from eating disorders, and, as a result, are best situated to understand the unique challenges they face in all aspects of their diagnosis, treatment and recovery)

^a^One such study already is underway led by Project HEAL and EAT Lab

^b^Notably, NCEED was designed to provide training and education for healthcare providers, trainees of all sorts, and even non-professional caregivers

*AED* Academy for Eating Disorders, *NCEED* National Center of Excellence for Eating Disorders, *MD* Medical doctors, *MSW* Masters of Social Work, *PhD* Doctorate of Philosophy, *RD* Registered Dietician, *REDC* Residential Eating Disorder Consortium

### Section III: standards of care

*The Status Quo* There are four categories of stakeholders in the field of eating disorders in the U.S.: advocacy organizations (Alliance, the National Association of Anorexia Nervosa and Associated Disorders [ANAD], the Eating Disorders Coalition for Research, Policy, and Action [EDC], the Eating Disorders Leadership Summit [EDLS], Families Empowered and Supporting Treatment for Eating Disorders [F.E.A.S.T.], the National Eating Disorders Association [NEDA], and Project HEAL), professional organizations (the Academy for Eating Disorders [AED] and the International Association of Eating Disorder Professionals [iaedp]), trade groups (the Residential Eating Disorder Consortium [REDC]), and educational and training groups (the National Center of Excellence for Eating Disorders [NCEED]). Each of these stakeholders has a significant interest in the standards used in regulating the diagnosis and treatment of eating disorders in the U.S. Those areas include: (1) national regulatory standards for the accreditation of eating disorders treatment facilities; (2) national accreditation of professionals specializing in the treatment of eating disorders; and (3) standards and guidelines for determining the type and level of care eating disorders patients receive.

- National Regulatory Standards for Eating Disorders Treatment Program Accreditation: The two prominent regulatory organizations in the U.S. are the Joint Commission on Accreditation of Healthcare Organizations (JCAHO) and the Commission on Accreditation of Rehabilitation Facilities (CARF). As a result of a multi-organizational task force comprised of representatives from AED, iaedp, and NEDA and led by AED, both JCAHO and CARF have adopted eating disorders specific criteria for inpatient, residential and partial hospital programs to be accredited as disease specific programs. These criteria are in need of continuous revision. Presently, however, there is not a dedicated resource within the field to monitor the criteria. One of the continuing gaps in criteria for being a specialized eating disorders program is how to assess the level of specialized competencies within all of the disciplines used in the treatment of eating disorders (e.g.*,* medical, psychiatric, psychotherapy, nursing, nutritional, and others).
- National Credentialing of Eating Disorders Professionals: The regulatory and insurance payors are progressively looking to the professional community for some credentialing mechanism that demonstrates that an individual has specialized training in the diagnosis and treatment of eating disorders. Currently, iaedp is the only professional organization in the U.S. that has created and offers an eating disorders specific certification process for various disciplines. Unfortunately, the iaedp credentialing process has not been consistently endorsed by other U.S. professional organizations.
- National Standards and Guidelines for Determining the Types and Levels of Care for Eating Disorders*:* There are multiple organizations, consortiums and industry groups that have issued guidelines relating to the treatment of eating disorders. Overall there is moderate to high consensus that several evidence-based treatments exist for outpatient treatment of AN, BN, and BED [8–10]. Unfortunately, there is less consensus regarding the best treatment strategies for eating disorders patients who do not remit with outpatient treatment. Historically, the American Psychiatric Association (APA) guidelines have been the gold standard for determining levels of care (outpatient, intensive outpatient, partial hospital, residential, inpatient) in the U.S. Unfortunately, these guidelines were suspended over the last several years and are currently undergoing revision. Although the APA guidelines were generally regarded as the gold standard, they have never been formally endorsed by all of the eating disorders organizations in the U.S.

*Consensus Point* Those afflicted with eating disorders, their loved ones, healthcare providers, and the eating disorders community as a whole would benefit from the establishment and maintenance of treatment program accreditation, professional credentialing, and treatment type and levels of care guidelines (Table 3).

**Table 3:** Goals and strategies for standards of care

Goal	Strategy
Both the JCAHO and CARF guidelines need to be continuously monitored and revised We need consensus regarding professional credentialing Ensuring that the soon-to-be published APA guidelines reflect the input of all stakeholders in the eating disorders field. Once the revisions to the APA guidelines are finalized, there needs to be a multi-organizational statement of support for the guidelines	We recommend that REDC establish a committee that maintains regular contact with these regulatory organizations. This committee should also explore how these organizations are assuring staff in these programs have specialized training in the diagnosis and treatment of eating disorders We urge the two predominant professional organizations (iaedp and AED) to collaborate on the process and content necessary for credentialing professionals. We recommend that iaedp include on its credentialing committee a member from the AED board to jumpstart the collaboration process. It is important that the agreed upon process and content be endorsed by both organizations The new APA guidelines, a working draft which is expected in Spring 2021, will have a substantial effect on how eating disorders care is delivered in the U.S. As the revision process unfolds, there will be some opportunity for interested parties to review and comment on the proposed changes. All stakeholders in the eating disorders field should embrace the opportunity to comment We propose that the EDLS, which consists of eating disorder organizations that represent the full range of disciplines and individuals (i.e., patients, carers) in the eating disorders field, spearhead the effort of drafting a multidisciplinary organizational statement of support for the APA guidelines resulting from the aforementioned process and build a consensus for issuance of that statement

*AED* Academy for Eating Disorders, *APA* American Psychological Association, *CARF* Commission on Accreditation of Rehabilitation Facilities, *EDLS* Eating Disorders Leadership Summit, *iaedp* International Association of Eating Disorder Professionals, *JCAHO* Joint Commission on Accreditation of Healthcare Organizations, *REDC* Residential Eating Disorder Consortium

### Section IV: research and research funding

*The Status Quo* Research funding for eating disorders is not commensurate with the severity of these illnesses. The federal funding allotted to eating disorders research in 2015 borders on the absurd—approximately $0.73 per affected individual [11]. By contrast, the federal government supported autism research at a per affected individual rate of $58.65, schizophrenia research at a rate of $86.97, and Alzheimer’s Disease research at a rate of $88 [12]. These figures are not offered to diminish in any way the severity of the latter diseases, but merely to highlight a gross disparity that has prevailed in the U.S. for decades where eating disorders are concerned. The figure associated with eating disorders research funding has decreased over time, given that, in 2011, it was $0.93 per affected individual [13]. Suffice it to say, there is only so much research progress one can expect with such limited resources.

Developing a career in eating disorders research is extremely challenging given the disparity between clinically relevant problems and research funding availability and priorities. As a result, the eating disorders field is hemorrhaging young eating disorder scholars. Moreover, researchers are striving to answer questions that have the greatest chance of being funded versus answering questions that are most important to the field. In other words, instead of science, clinical experience, and patients’ needs driving science aimed at creating clinical impact, money is driving the science because researchers are scrambling to keep their jobs. Further, under conditions of scarce resources, it becomes harder for science to self-correct because (a) people find contrary findings threatening and (b) it is extremely hard to switch research programs if one’s original research hypotheses were proven incorrect. In other words, because switching programs of research is exceedingly difficult, researchers are incentivized to design studies aimed at supporting their model or treatment as opposed to identifying when a treatment or model fails, even though we need failures to move science forward. Scarce resources also limit data sharing, open science, replication, and reproducibility.

There are bottlenecks that hamper the development of new eating disorder researchers. On the positive side, there are an increasing number of eating disorder experts available to train new eating disorder researchers in clinical psychology doctoral programs. On the negative side, we cannot expect this trend to be maintained because obtaining a faculty position at a major research university in clinical psychology has become increasingly difficult. Thus, many newly trained psychology scholars are taking positions with higher undergraduate teaching loads, which reduces research productivity. In addition, fewer academic medical centers offer training to physicians (including psychiatrists), medical students, and allied health professionals and students in eating disorders care and research.

Moreover, the changes in academic medical centers have impacted opportunities for clinical psychologist training at the internship level, driving promising young scholars away from the field [7, 14]. One key driver of the changes occurring in academic medical centers is that eating disorder care is neither as profitable as other forms of medical care (e.g., bariatric surgery), nor as likely to result in research money given the limited National Institutes of Health (NIH) expenditures in this area. In addition, patients with insurance are increasingly seeking care at for-profit treatment centers, reducing the availability of patients to serve both as research participants and to help educate the next generation of clinicians about eating disorders. From the research side, obtaining the sample sizes needed for definitive research is difficult. From the clinician side, there is a shortage of physicians, psychologists, and therapists adequately experienced and trained in the assessment and treatment of eating disorders.

With regard to nutrition research, there is an extreme shortage of quality research. Most research focuses on “concerns about obesity,” and almost none of this research investigates negative outcomes with regard to eating pathology. The bariatric surgery literature similarly fails to adequately address eating pathology.

*Consensus Points* The workgroup identified several Consensus Points as below:

- The establishment and implementation of effective, empirically/evidence-based standards of care requires research across a broad spectrum of domains (e.g., epidemiology, genetics, neurobiology, nutrition, medicine, behavior, psychology, sociology, neuroscience), a diverse range of populations, adequate private and government funding, and the free exchange of ideas and information among all who share a commitment to understanding, treating, and, ultimately, markedly diminishing the negative impact of eating disorders.
- The “eating disorder stereotype” has limited the field’s definition of eating disorders and eating disorders research. It also limits the perceived public health impact of eating disorders, impacts perceptions of who gets diagnosed with an eating disorder, and contributes to the perception that “disordered eating” and eating disorders are fundamentally different (versus representing different points on a spectrum of eating behavior ranging from normal/healthy to extremely pathological). This has led to barriers and delays in providing care related to eating behaviors and cognitions. One first step in improving the eating disorders field with respect to research and funding is to reclassify eating disorders as eating spectrum disorders (ESD) to encompass the full spectrum of eating pathology.
- The eating disorders research field has historically been criticized for being insular. The field would benefit from greater participation in wider mental health research at all levels (conferences, leadership in generalist mental health organizations, publication in generalist journals, participation in generalist editorial boards and NIH study sections; regular dialogue with the US-based Centers for Disease Control and Prevention).
- Although NIH institutes that target “medical” conditions are increasingly funding research studying behavioral interventions, the National Institute of Mental Health (NIMH) has moved in the opposite direction and is largely the institute of neuroscience. Support for foundational research that has led to major treatment successes (dialectical behavioral therapy, family-based therapy, cognitive-behavioral therapy for a range of disorders) is significantly more difficult to obtain from NIMH, given the increased focus on biological aspects of mental health. We need an institute (or other funding mechanism) that funds behavioral science in the area of mental health. This would also facilitate the study of combined behavioral and biological interventions. Such an endeavor should be taken on by more than just the eating disorders field (e.g., partner with the Coalition for the Advancement and Application of Psychological Science). Importantly, the lack of funding is driving promising and sorely needed junior researchers out of the field into clinical jobs.
- Seven key limitations in the research environment must be addressed
  - Researchers and clinicians need greater respectful collaboration in identifying and addressing clinically relevant questions. This could potentially be self-funded by treatment centers, bypassing the NIMH problem.
  - We have insufficient, understandable research addressing problems in the dissemination and implementation of our existing effective treatments. Although NIMH has a funding mechanism for dissemination and implementation research, this mechanism is designed to advance dissemination and implementation science, which is aimed at big-picture, cross-cutting dissemination and implementation questions. This poses two problems for the eating disorders field. First, dissemination and implementation science is extremely jargon heavy and aimed at the large-scale questions, meaning that many of its findings are hard (if not impossible) to translate into easy to understand, practical solutions for specific problems. Second, the funding mechanism is not intended to answer any questions that are very specific to one type of setting, disorder, and/or treatment. For this reason, this research for eating disorders will need to be funded outside NIMH’s dissemination and implementation funding stream to address eating disorder specific questions.
  - We need significant expansion of research studying clinically relevant questions with diverse populations to understand to what degree treatments that were developed with predominantly white, female populations can be applied (or need to be modified) to meet the needs of all people who struggle with eating pathology.
  - We need increased research investigating how to translate nomothetic treatments (i.e., treatments developed based on averages) into idiographic (i.e., treatments developed and personalized based on the individual) evidence-based treatment.
  - We need to make it easier to present and publish negative findings.
  - Eating disorders researchers should be encouraged to freely share pre or post prints so that clinicians and service users may have unrestricted access to the research.
  - We need increased research on low-cost, scalable interventions and to study novel strategies aimed at creating broad public health impact (Table 4).

**Table 4:** Goals and strategies for research and research funding

Short-term goals	
*Change conceptualization of eating disorders* • Introduce and begin to validate the concept of eating spectrum disorders (ESD); encourage researchers to consider what full dimensional classification of eating pathology would look like. This would include research on symptom-based classification and the interaction of symptoms with treatment • Challenge categorical distinctions (e.g., disordered eating vs. eating disordered; recovered, partially recovered, not recovered; AN binge/purge vs BN; binge-eating with dieting vs atypical AN; AN, restrictive with low insight vs ARFID) and work toward dimensional assessment of these outcomes • Advocate/lobby that eating disorder cognitions and behaviors be assessed in current studies examining other psychiatric patient populations such as mood, anxiety, and substance use disorders. Currently, we believe that because eating disorders are generally only evaluated and considered by researchers within this field, the impact of ESD on other mental illnesses is missed. This could look like a supplement for existing NIMH grants, and would be particularly helpful if targeted to existing large-scale studies in addictions and mood disorders *Bridge the clinical-research gap* • Develop a menu of standardized self-report measures that are routinely used pre/post and, optimally at follow-up across treatment centers and with other providers of ESD care. Suggested possible measures: EDE-Q [17], PHQ-8 [18], GAD-7 [19], demographics, weight, height. Recording if patient aware of weight or not for any clinical treatment setting or study. This development should include exploration of existing and past initiatives, including the NIH Assessments/Toolkit for Eating Disorders *Answer fundamental questions* • Expected treatment course/symptom fluctuations • How does clinical course vary based on the specific ESD diagnoses vs. clinical symptoms? • Tracking eating symptoms amongst the majority of people with eating disorders that never need intensive/inpatient care for an eating disorder • Determine when it is appropriate to transition between levels of care and how long is needed for an appropriate course of treatment *Improve dialogues between clinicians and researchers* • Provide pre/post prints freely available to clinicians • Link the annual EDRS and ICED meetings to improve attendance at both and allow researchers to attend more generalist and/or related specialty conferences. Linking EDRS and ICED (e.g., have EDRS precede ICED in the same location) will reduce both the costs and carbon footprint for those who attend both conferences, as well as free up time *Improve attention to issues of diversity in ESD research* • Ask ESD journal editors to require that all studies report a full breakdown of race/ethnicity, gender identity, and socio-economic status • Replicate existing findings in diverse populations • Create library of results needing replication or extension into other populations • Offer mentorship through AED or EDRS to help scholars frame replication studies that are adequately powered and designed to confirm or refute initial study findings • Encourage researchers to start studying low-cost, scalable interventions in conjunction with clinician networks	
*Improve attention to issues of diversity in ESD research* • Ask ESD journal editors to require that all studies report a full breakdown of race/ethnicity, gender identity, and socio-economic status • Replicate existing findings in diverse populations • Create library of results needing replication or extension into other populations • Offer mentorship through AED or EDRS to help scholars frame replication studies that are adequately powered and designed to confirm or refute initial study findings • Encourage researchers to start studying low-cost, scalable interventions in conjunction with clinician networks *Accept comorbidity as norm in ESD* • Move into more consistent dimensional assessment of eating pathology in conjunction with tracking anxiety, depression, and substance use disorders • Work with NIH to add funding mechanisms that support collection of eating pathology data for existing studies of depression, anxiety and substance use disorders • Broaden our engagement with NIH study sections and staff (e.g., identify study sections that are more amenable to investigation of comorbidity and dimensional assessment so that such studies can be routed to these study sections) • Educate NIH reviewers to accept real patients rather than perfect patients without comorbidities, as well as patients without a ‘strict’ diagnosis. Disseminate information to eating disorder researchers about NIH study sections that welcome and/or are open to dimensional approaches to eating disorders and those that model comorbidity. Some example NIH study sections include BRLE, BGES, PDRP *Retaining/building new researchers in ESD and reducing insularity* • Educating researchers at conferences on how to get papers published in generalist journals • Educating researchers on how to review for generalist journals • Approach editors of key journals about initiatives to publish both negative and replicated findings • Begin creating an action plan for a new NIH institute focused on behavioral science in the area of mental health	

Long-term goals	
• Change DSM-5 from Eating and Feeding Disorders to ESD, or alternative conceptualization that can cover all types of eating disorder behaviors and related cognitions *Create centralized ESD research consortium* • Input Clinical Data—programs, outpatient clinicians, or patients themselves could send standardized data (5 or 6 recommended measures) at regular intervals creating access to standardized and large datasets (i.e., big-data) to answer relevant clinical questions • Individual researchers can sign onto bigger projects • Commitment to funding a larger range of eating disorder researchers so that we broaden the researcher base and bring more creativity to the table *Establish key measures in assessment of ESD* • Identify other alternatives for determining “health” instead of weight/BMI (e.g., Total T3; Leptin) and determine when focus should be on weight and BMI in addition to other metrics. Ensure (and develop) metrics for determining “health” that are appropriate for diverse and underrepresented persons • Bridge and engage with obesity research to ensure assessment of eating disorder behaviors in their research. While we recognize there may be concerns about these collaborations, to strengthen the science of eating disorders, as well as decrease weight stigma and biases in the obesity field, the best approach will be collaborative, in which we draw from the ‘best’ of each field, such that both fields can benefit mutually from each other *Expand funding base* • Challenge funding sources to move away from categorical diagnosis • Create new sources of funding that will let science and clinical questions drive science (as opposed to NIMH funding priorities) • Create a new NIH institute or alternative funding mechanism at a similar level to address the consensus research points • Find ways to use CMS) database to promote evidence-based outpatient care *Broaden base of ESD researchers* • Identify generalist journals that need or could benefit from ESD aware professionals on their editorial boards; develop a plan to get those representatives on the boards • Support movement of researchers in ESD into and back from other broader areas (e.g., anxiety, depression, behavioral genetics); encourage researchers in other areas (mood, trauma, addiction) to conduct studies in ESD and support those researchers to obtain publications/grants in ESD	

Strategy	Obstacles	Navigation
Build support for ESD by: • Conducting a literature review (and/or meta-analysis) to set the stage for discussion • Encourage researchers to collect data to create an empirically supported dimensional classification system for ESD • Obtaining support of major players: APA (for DSM), AED, CMS, NIH, NEDA, iaedp, residential treatment programs (both for- and not-for profit), and HiTOP • Work with EDRS and AED to build support for a combined meeting • Work with REDC, AED, iaedp, NEDA, and treatment centers to begin standardization of measures and open publishing of outcome data to create a centralized EDS research consortium • Build or enhance workshops in iaedp, EDRS, AED, etc. on team science and collaboration across treatment centers, medical providers, and scientists to achieve united goals • Work with conferences and organizations to create education and training for researchers and trainees on how to obtain ESD funding, how to be on NIH study sections/identify study sections appropriate for one’s work, how to identify program officers whose programs fund eating disorders work (e.g., Janani Prabhakar, Mark Chavez, Julia Zehr, Mary Rooney), publish in more journals, and how to do open science	Change is hard. People like the status quo Retraining/re-educating on ESD may be needed Some may be committed to the existing but narrow definitions of AN/BN/BED The lack of funding and financial prioritization available to create and build these initiatives If the field does not grow by inviting others in, then slices of the pie will be too small for those here now Tensions within the field between academic and for-profit treatment centers	Identify concerns and obstacles Lobby players to support conceptualization Develop new funding streams to support innovative/spectrum approaches (i.e., invite and pay researchers to join ESD consortium standardization for big clinical questions) Collaborate with members of the ESD field who have tried to accomplish some of these goals in the past to learn from their experiences

*AN* anorexia nervosa, *AED* Academy for Eating Disorders, *ARFID* avoidant/restrictive food intake disorder, *BGES* behavioral genetics and epidemiology, *BN* bulimia nervosa, *BED* binge-eating disorder, *BMI* body mass index, *BRLE* biobehavioral regulation, learning, and ethology, *CMS* Centers for Medicare and Medicaid Services, *DSM-5* Diagnostic and statistical manual of mental disorders; fifth edition, *EDE-Q* eating disorders examination questionnaire, *EDRS* eating disorders research society, *ESD* eating spectrum disorders, *GAD-7* generalized anxiety disorder-7, *ICED* international conference on eating disorders, *NIH* National Institutes of Health, *NIMH* National Institute of Mental Health, *PDRP* psychosocial development, risk, and prevention, *PHQ-8* patient health questionnaire-8

### Section V: advocacy, education, and legislation

*The Status Quo* There are a number of organizations in the eating disorders community whose Mission Statements include and whose leadership and membership groups have long been committed to: (1) promoting state and federal legislative initiatives relating to eating disorders research, training, treatment, and awareness; (2) advocating on behalf of eating disorders sufferers with respect to issues including early intervention, greater accessibility to affordable, evidence-based care, and enhanced insurer reimbursement for treatment; and (3) educating parents, students, teachers, coaches, and frontline health care providers on best practices relating to the early detection, treatment, and risks associated with these life-threatening illnesses.^2^

There also are dozens of websites, webpages, and social media based private and public groups, whose participants serve as zealous advocates, offer peer-to-peer and/or professional support, and provide educational resources on behalf of those who are battling or in recovery from eating disorders and the loved ones committed to supporting them, as well as those seeking to learn more about these often overlooked and frequently misunderstood illnesses.^3^ Finally, there are countless individuals with lived experience, tech savvy clinicians, bloggers, and others who consistently use their voices and platforms to raise awareness, promote education, and actively lobby on all matters eating disorder related.

Despite the selfless and tireless efforts of these individuals and organizations, however, federal and state governments have been slow to take a proactive role in addressing the myriad needs confronting the eating disorders community. Indeed, notwithstanding the fact that eating disorders advocates have been aggressively pursuing federal legislative assistance since the introduction of the Federal Response to Eliminate Eating Disorders Act (FREED Act) in 2009^4^ and introduced similar legislation again in 2011,^5^ 2013^6^ and 2015^7^; it wasn’t until December, 2016, when President Obama signed the twenty-first Century Cures Act into law that the words “eating disorders” first appeared in a piece of enacted federal legislation in the U.S.

Make no mistake, that legislation is significant in that it: (1) clarifies that insurance coverage of eating disorders treatment is subject to the parity provisions of the Mental Health Parity and Addiction Equity Act (MHPAEA); and (2) articulates the need for and plans to better educate medical professionals and the general public about early identification of eating disorders [15]. However, there is considerable work to be done in advancing and funding the ground-breaking research and other educational initiatives that were integral pieces to the FREED Act and addressing the plethora of other needs confronting those suffering from and of those who have dedicated their professional lives to better understanding and treating these illnesses.

*Consensus Point* When it comes to core issues affecting all sufferers of eating disorders (e.g., benefit of early intervention; reasonable accessibility to evidence-based care; quality and affordability of care; need for research, increased public awareness and support, and legislative initiatives) those afflicted with eating disorders, their loved ones, and the eating disorders community as a whole would benefit from speaking with a unified voice (Table 5).

**Table 5:** Goals and strategies for advocacy, education, and legislation

Short- and long-term goals	
*Prioritize consensus building* As the autism experience dramatically illustrates, the ability to unify (i.e., reach a consensus) and speak with a singular voice significantly enhances the likelihood of achieving the legislative, funding, and educational objectives of those who share a common enemy (e.g., autism)—and the same is true of eating disorders. Conversely, speaking with a splintered voice makes it difficult for those who are in a position to legislate, fund, and/or otherwise effect meaningful change to identify and respond to core issues and needs *Develop carefully tailored messaging* Word selection, message framing, and a clear understanding of and sensitivity to the intended audience are critically important to being heard and achieving desired results in the legislative, corporate, academic, and public arenas that are indispensable to the achievement of the eating disorder community’s goals. The same is true with respect to the individual decision-makers and decision-influencers who are the intended and/or likely recipients of that messaging *Develop and work from a common set of data* Advocacy, education, and legislative and funding initiatives are much more impactful if they are grounded in empirical data that is credible and readily defensible. For too long, the eating disorders community has been reliant on incomplete, anecdotal, and/or inconclusive data that only serves to: confuse, if not distort its intended messaging; convey a sense of disorganization; diminish the credibility of the community as a whole; and detract from the gravity of the situation *Make more effective and concerted use of technology* The proliferation of social media platforms provides the eating disorders community with a ready and cost-effective means of reaching tens, if not hundreds of thousands of individuals and organizations from a single laptop in a matter of minutes. Exploring creative ways of harnessing and maximizing the use of these currently underutilized resources to further educational, advocacy, and legislative initiatives is and, in the years to come, will be critical to their success *Open cross-disciplinary lines of communication* Two of the take-aways from the Summit were: (1) the well-spring of ideas that can come from providing a space in which diverse members of the eating disorder community (e.g., researchers, clinicians, academicians, advocates, people with lived experience, and family members) can freely express their thoughts; and (2) a sense of regret that there are too few opportunities to do so. Meaningful progress depends on making such cross-disciplinary exchanges (real or virtual) the rule, rather than the exception *Redouble efforts relating to diversity and inclusion* There is a growing awareness that issues related to racial, ethnic, size, and cultural diversity, as well as gender identity and sexual orientation, have a significant impact on all aspects of an individual’s diagnosis, treatment, and recovery from an eating disorder. To the community’s credit, progress has been made when it comes to embracing and attempting to rectify those disparities. However, there is much work left to be done to reshape and refocus the predominate lenses through which these illnesses historically have been viewed to ensure that diverse populations gain increased visibility *Return to our collective roots* It is easy given the busyness and daily demands of life and the often soul-depleting nature of eating disorders for those charged with advocating, educating, and/or promoting legislative initiatives on behalf of those who suffer from eating disorders to lose sight of the fact that we are fighting a common enemy (eating disorders) and are committed to a common goal (making quality care accessible and affordable to all eating disorders sufferers and working towards the eventual eradication of those illnesses). The same is true for those whose lives have been touched by eating disorders and who have made the study and/or treatment of eating disorders their life work. In that fight, solidarity should be our guiding principle	

Strategies for achieving goals	
*Consensus Building* Convene a virtual summit meeting of representatives of the leading eating disorder advocacy organizations and stakeholders for a twofold purpose: To commit to speak with a singular voice on core issues and emergent needs facing all eating disorder sufferers, including unique considerations of race, gender identity, sexual orientation, ethnicity, size, and/or age (e.g., the accessibility, availability and affordability of care; the need for evidenced-based standards of care; the need for more robust research and research funding; and the need for adequate and equitable treatment from insurers) To reach a consensus on a specific platform of messaging that is fact based, data supported, narrowly tailored to the intended audience(s), apolitical, capable of ready adaptation to all forms of social, print, and video media, and highly compelling, together with a corresponding commitment that each stakeholder will push the messages out—consistently and enthusiastically—cognizant of the fact that doing so benefits all sufferers *Tailored messaging* It is impossible to control, nor should any effort be made to control, the messages individuals choose to post on their social media platforms relating to eating disorders. However, those organizations who serve as the faces and voices of the eating disorder community as a whole have a heightened responsibility to ensure that the messages they create and promote are evidence-based, carefully framed, and reflect a clear understanding of and sensitivity to their intended audience(s) (i.e., those who are likely to “consume” them) Those audiences include: state and federal legislators, corporate executives, insurance company representatives, frontline physicians and clinicians, private foundations, and wealthy individual benefactors—many of whom lack even a fundamental understanding of eating disorders and their life-threatening nature. They do, however, tend to be highly sophisticated and to have certain expectations with respect to advocacy and messaging when it comes to groups and individuals vying for their attention, their monetary and policy support and/or philanthropy—all of which are critical to the achievement of the eating disorder community’s goals. That being the case, that messaging cannot be relegated to those who lack the experience, discretion, sophistication, and communication skills required to maximize the likelihood that it will be heard and favorably acted upon. Instead, ideally, those charged with advocacy, education, and/or advancing legislative initiatives in the eating disorders community should retain and rely on professional publicists, media consultants, and marketing firms to assist them in formulating, tailoring, and properly disseminating the critical messaging referenced in the preceding point *Help facilitate the timely and efficient dissemination of critical research findings and data* Eating disorder education and advocacy, as well as the promotion of legislative initiatives aimed at advancing the needs of eating disorder sufferers stand to benefit significantly from ground-breaking research, including the initiatives outlined in this Report, as well as the remarkable work that already has been and is being done in the U.S. and in a myriad of other countries around the world who are battling these insidious illnesses. The recent publication of the AED/STRIPED economic impact study is a prime example, especially with respect to identifying the estimated societal, health care, and personal economic costs of eating disorders in the U.S. However, the impact of these and other initiatives is only as great as the mechanisms that exist to facilitate its prompt and efficient dissemination to organizations and individuals who are in a position to make effective use of it in furtherance of its intended purposes and to effect change (i.e., legislators, corporate executives, insurance company representatives, frontline physicians and clinicians, elementary and secondary educators and administrators, private foundations, and wealthy individual benefactors). With proper guidance from researchers, those in the advocacy, education, and legislative initiative(s) community and their established distribution networks could be uniquely situated to assist in those efforts^a^	
*More effective use of technology* The importance of the effective, concerted, consistent, and proper use of technology to the achievement of eating disorder advocacy, education, and legislative goals cannot be overstated and, therefore, must be spearheaded by highly-qualified and highly-skilled professionals, especially as it relates to core (consensus based) messaging. That can be accomplished in one of several ways First, the leading eating disorder advocacy organizations and stakeholders, working from a place of consensus, could contribute on a pro rata basis towards the retention of a full-time expert in the field to design and establish a game plan for implementing a comprehensive strategy for efficiently and cost-effectively disseminating critical messaging to the target audiences^b^. Alternatively, NCEED could designate such an expert, who could be informed by the aforementioned stakeholders, since it already has ties to the target audiences and access to the requisite channels Second, if budgetary constraints make full-time employment impossible, secure the services of the aforementioned expert for purposes of designing the strategy and training hourly employees and interns in each of the member organizations to execute that plan with the understanding that the consultant will be available on an as needed basis Third, implement policies and procedures and, carefully, monitor compliance to ensure that those who have the access/authority required to post on the organization’s social media and other internet platforms understand what is (and is not) permitted in the way of messaging as it relates to core issues impacting all eating disorder sufferers *Cross-disciplinary lines of communication* In addition to annual or semi-annual conferences at which stakeholders or their designees deliver formal presentations to other stakeholders, eating disorders community organizers and sponsors should focus on arranging in-person or virtual conferences aimed at promoting the free exchange of ideas and information among multi-disciplinary professionals with an eye towards: fostering more open lines of communication among the various disciplines; identifying and prioritizing gaps and goals needed to advance the core objectives of the eating disorders community as a whole; relationship and consensus building; providing opportunities for the next generation of treatment providers to benefit from the knowledge and wisdom acquired by long time leaders and practitioners in the field; affording those charged with advocating, educating, and advancing legislative initiatives with a broad-based understanding of cutting-edge developments and research in the field to further enhance their efforts. Simply put, where communication is concerned, the eating disorder community needs to reach beyond those who already appreciate the seriousness of these illnesses. It is time to shift the focus and the messaging (in a more concerted way) to those outside the eating disorder community, who not only remain largely underinformed about the magnitude and gravity of the problem, but in many instances are uniquely situated to be instrumental in effecting meaningful change once provided with a clear understanding of what change is needed^c^	
*Greater diversity and inclusion* Eating disorders do not discriminate, yet if the eating disorders community were to take an objective snapshot of the current landscape, it also would be forced to admit that those differences are not adequately represented in positions of influence in the eating disorder field. It is up to the eating disorders field to be intentional in reconstituting its own house to reverse that state of affairs. As importantly, it is undeniable that being different (i.e., not fitting the stereotypic mold of an eating disorder sufferer—affluent (or at least reasonably well insured), well-educated, white female) makes a difference when it comes to: the likelihood of being properly and timely diagnosed; the availability, accessibility, affordability, and quality of care; the chance of being treated by a provider who is sensitive to racial, ethnic, gender identity, sexual orientation, and/or cultural dynamics and/or nuances that may influence the recovery journey; and post-treatment support communities comprised of like-experienced and/or like-minded peers. That too, needs to change and the advocacy stakeholders can take a powerful and lead role in educating and advocating for that change *Back to basics* Few professional and advocacy communities are populated with individuals and organizations whose fundamental purpose is (or should be) to one day “put themselves out of business,” but the field of eating disorders is one of them. And, ideally, one day that will happen (i.e., because of our collective efforts, eating disorders will be eradicated or their threat so minimal as to only need a fraction of the resources currently dedicated to fighting them). In the interim, however, all who warrior against these illnesses, especially those charged with advocating, educating, and/or promoting legislative initiatives on behalf of all those who suffer from eating disorders must not lose sight of the fact that: (1) we are all in this (and stronger) together; and (2) solidarity is where each of our journeys began and it is the light that will illuminate the path forward	

^a^NCEED is one avenue that may be particularly effective for dissemination of research findings and data given its ties to federal partners and its collaborative relationships across academic medicine, organizations represented in REDC, and advocacy/policy groups like the Eating Disorders Coalition, NEDA, Project HEAL, and The Alliance for Eating Disorders

^b^NCEED is already poised to do this work (and funded to do so). It would welcome the opportunity to work from a place of consensus with the larger field to help disseminate critical messaging to various stakeholder audiences. And to the larger point re: needing professional help to do so. In fact, NCEED has hired a communications firm with experience in health communications to help achieve KPIs and we could easily incorporate this work into those KPIs

^c^NCEED is situated to have a crucial impact here. Since its inception, NCEED has pivoted its educational and training efforts to focus on primary care/frontline clinicians (with a still present but less emphasized effort on stakeholders who already constitute the eating disorders field). Given its established relationships with stakeholders outside the eating disorder community, NCEED is a natural fit for fostering these lines of communication

*AED* academy for eating disorders, *NCEED* National Center of Excellence for Eating Disorders, *STRIPED* strategic training initiative for the prevention of eating disorders

## Conclusions

The Summit culminated in a Report which serves as the basis of this manuscript. The authors agree that there are many finer points that merit further consideration and details that need to be fully elucidated in order to enact any of the proposed recommendations. However, there was broad-based consensus on the below points regarding the eating disorders field and how best to advance it.

- Eating disorder outcomes and prevention efforts can be improved by implementing creative health education initiatives that focus on societal perceptions, early detection, and timely, effective intervention. Such initiatives should be geared toward parents/guardians, families, other caretakers, and frontline healthcare providers in order to maximize impact.
- Those afflicted with eating disorders, their loved ones, and the eating disorders community as a whole would benefit from greater accessibility to affordable quality care, as well as greater transparency and accountability on the part of in-hospital, residential, and outpatient health care providers with respect to their qualifications, methodologies, and standardized outcomes.
- Those afflicted with eating disorders, their loved ones, health care providers, and the eating disorders community as a whole, would benefit from the establishment and maintenance of treatment program accreditation, professional credentialing, and treatment type and levels of care guidelines as has been outlined by groups of eating disorder professionals outside the US [16].
- The establishment and implementation of effective, empirically/evidence-based standards of care requires research across a broad spectrum of domains (e.g., epidemiology, genetics, neurobiology, medicine, behavior, psychology, sociology, neuroscience), a diverse range of populations, adequate private and government funding, and the free exchange of ideas and information among all who share a commitment to understanding, treating, and, ultimately, markedly diminishing the negative impact of eating disorders.
- The “eating disorder stereotype” has limited the field’s definition of eating disorders and eating disorders research. It also limits the perceived public health impact of eating disorders, impacts perceptions of who gets diagnosed with an eating disorder, and contributes to the perception that “disordered eating” and eating disorders are fundamentally different (versus representing different points on a spectrum of eating behavior ranging from normal/healthy to extremely pathological). This has led to barriers and delays in providing care related to eating behaviors and cognitions.
- One first step in improving the eating disorders field with respect to research and funding is to reclassify eating disorders as ESD to encompass the full spectrum of eating pathology.
- The eating disorders research field would benefit from greater participation in wider mental health research at all levels (conferences, leadership in generalist mental health organizations, publication in generalist journals, participation in generalist editorial boards and NIH study sections; regular dialogue with CDC).
- Several key limitations in the eating disorders research literature must be addressed.
- When it comes to core issues affecting all sufferers of eating disorders (e.g., the benefit of early intervention, reasonable accessibility to evidence-based care, the quality and affordability of care, the need for research, increased public awareness and support, and legislative initiatives) those afflicted with eating disorders, their loved ones, and the eating disorders community as a whole would benefit from speaking with a unified voice.

*Limitations* The authors readily acknowledge that there is room to disagree over a word, a phrase, a sentence or, perhaps, even a recommendation (or two) in this paper. They also recognize that not everyone in the field will necessarily agree with every recommendation in this paper. Indeed, some people who agreed with some sections chose not to endorse the Report and Recommendations upon which it is predicated because they also disagreed with other sections. In such cases, we hope that people will nonetheless come together where they find agreement.

Another limitation is that this paper and the underlying Report are (by design) U.S.-Centric. Consequently, while it is it is likely that many recommendations will benefit the global eating disorders field, not all will. In addition, although an effort was made to include a wide range of constituents during the Summit, a decision was made to limit the number of participants to around 25 so that the number of individuals did not become unwieldy. By definition, this means that not everyone who is a member of our field, including, but not limited to, those from other countries, will see themselves represented in the authors. This too is a limitation. As noted earlier, however, we view this paper and the Report on which it is predicated as a first step, not a final step in generating collaboration.

The authors also acknowledge that there is still much work to be done when it comes to tackling the myriad of issues confronting the diverse needs of those caught in the grip of these insidious illnesses. However, given the gravity and urgency of the situation and the preciousness of the lives hanging in the balance, the consensus among the Summit participants is that: (1) the status quo is unacceptable; (2) the need for a thoughtful and unified plan of action is immediate; and (3) the time for meaningful progress is long overdue. Thus, the Summit participants’ hope is that this paper and the Report on which it is predicated will serve as a catalyst for further consensus-building and a blueprint for hope and healing for years to come.^8^

## Data Availability

Not applicable.

## Abbreviations

ACCME: Accreditation Council for Continuing Medical Education
AED: Academy for eating disorders
AN: Anorexia nervosa
ANAD: National Association of Anorexia Nervosa and Associated Disorders
APA: American Psychological Association
ARFID: Avoidant/restrictive food intake disorder
BED: Binge-eating disorder
BGES: Behavioral genetics and epidemiology
BMI: Body mass index
BN: Bulimia nervosa
BRLE: Biobehavioral regulation, learning, and ethology
CARF: Commission on accreditation of rehabilitation facilities
CMS: Centers for Medicare and Medicaid Services
CDC: Centers for disease control
DSM-5: Diagnostic and statistical manual of mental disorders, fifth edition
EDC: Eating disorders coalition for research, policy and action
EDLS: Eating disorders leadership summit
EDE-Q: Eating disorder examination-questionnaire
EDRS: Eating disorder research society
ESD: Eating spectrum disorders
F.E.A.S.T: Families empowered and supporting treatment for eating disorders
FREED Act: Federal response to eliminate eating disorders act
GAD-7: Generalized anxiety disorder-7
HiTOP: Hierarchical taxonomy of psychopathology
Iaedp: International Association of Eating Disorder Professionals
ICED: International conference on eating disorders
JCAHO: Joint Commission on Accreditation of Healthcare Organizations
MHPAEA: Mental Health Parity and Addiction Equity Act
NASMHPD: National Association of State Mental Health Program Directors
NCEED: National Center of Excellence for Eating Disorders
NEDA: National Eating Disorders Association
NIH: National Institutes of Health
NIMH: National Institute of Mental Health
PDRP: Psychosocial development, risk, and prevention
PHQ-8: Patient health questionnaire-8
REDC: Residential eating disorders consortium
US: United States
YRBS: National youth risk behavior survey

## Footnotes

1 While the substance of the Report and Recommendations has been preserved and is presented herein, certain changes in formatting and wording were made to the original Report to conform to publication standards. A copy of the original Report and Recommendations can be found at https://osf.io/bh94w/.

2 Those organizations include, among others: the National Eating Disorders Association (NEDA), the Academy for Eating Disorders (AED), The Alliance for Eating Disorders Awareness, the Eating Disorders Coalition (EDC), Families Empowered and Supporting Treatment for Eating Disorders (F.E.A.S.T.), the International Association of Eating Disorders Professionals (iaedp), the National Association of Anorexia Nervosa and Associated Disorders (ANAD), Project HEAL, the Multi-Service Eating Disorders Association (MEDA) and the Residential Eating Disorders Consortium (REDC).

3 Some of those resources include: ATDTfb—Eating Disorder Family and Carer Support (https://www.facebook.com/groups/ATDTCarerSupportGroup); Mothers Against Anorexia Nervosa and Bulimia Nervosa (https://www.facebook.com/Mothers-Against-Anorexia-and-Bulimia-1578282975793738); Eating Disorder AN, BED BN & EDNOS Recovery Support Group (https://www.facebook.com/groups/edrecoverymentalhealthsupport); Sockit To ED https://www.facebook.com/groups/SockitToEDglobal); and World Eating Disorders Day (https://www.facebook.com/WorldEatingDisordersDay)—to name only a few.

4 If passed, that Act would have required the National Institutes of Health (“NIH”) to take myriad steps, the most notable of which included: (1) compiling statistics on the economic cost of eating disorders; (2) consulting with eating disorder researchers to implement a comprehensive long term plan for research on eating disorders; (3) annually submitting to Congress a scientifically-justified budget on eating disorders research; (4) establishing “Centers of Excellence” for the purpose of training researchers and conducting research; and (5) creating a clearinghouse for eating disorder research information at the Center for Disease Control and Prevention.

5 Like its predecessor, the 2011 version of the Act contemplated sweeping initiatives coordinated through NIH in the areas of: (1) eating disorder research aimed at identifying and classifying eating disorders, ferreting out the causes of eating disorders and establishing guidelines for their diagnosis, early detection, and treatment; (2) the development and evaluation of new treatment protocols and best practices; (3) a comprehensive assessment of existing eating prevention programs and the development of reliable prevention and screening programs; (4) a strategic plan for the conduct of, and support for, eating disorder research, including proposed budgetary recommendations, an award of federal grants for the purpose of improving the collection, analysis and reporting of State epidemiological data on eating disorders, and a joint study on the impact eating disorders have on educational advancement and achievement beginning in elementary schools.

6 The 2013 Act had a heavy emphasis on research aimed at better understanding, diagnosing, and treating eating disorders and how to more quickly identify and intervene in them in the lives of those afflicted.

7 The 2015 bill, dubbed the Anna Westin Act, marked a notable shift in focus away from research and collaboration. Instead, broadly construed, it sought “clarification” that, pursuant to the Mental Health Parity Act of 2008 (“MHPAEA”), a group health plan or policy that afforded coverage for eating disorders necessarily had to include residential treatment.

8 Notably, the Summit participants were asked to contribute a “Legacy List” of 10 of the most important lessons, pieces of advice, or practice tips relating to eating disorders that they’ve learned, been given, or used in the course of their work in the field. Those lists will be collated and published at a later date as a stand-alone resource.
